# Genome Sequence of the Yeast *Cyberlindnera fabianii* (*Hansenula fabianii*)

**DOI:** 10.1128/genomeA.00638-14

**Published:** 2014-08-07

**Authors:** Kelle C. Freel, Véronique Sarilar, Cécile Neuvéglise, Hugo Devillers, Anne Friedrich, Joseph Schacherer

**Affiliations:** a Department of Genetics, Genomics and Microbiology, Université de Strasbourg/CNRS, UMR 7156, Strasbourg, France; b INRA, UMR 1319 Micalis, Bât. CBAI, Thiverval-Grignon, France; c AgroParisTech, UMR 1319 Micalis, Bât. CBAI, Thiverval-Grignon, France

## Abstract

The yeast *Cyberlindnera fabianii* is used in wastewater treatment, fermentation of alcoholic beverages, and has caused blood infections. To assist in the accurate identification of this species, and to determine the genetic basis for properties involved in fermentation and water treatment, we sequenced and annotated the genome of *C. fabianii* (YJS4271).

## GENOME ANNOUNCEMENT

The species *Cyberlindnera fabianii* (1) (previously *Hansenula fabianii*, *Pichia fabianii*, and *Lindnera fabianii*) is an ascomycetous yeast in a clade of 27 species (2). Ascospore morphology varies across the clade and some species are heterothallic (2). *C. fabianii* has been used to treat wastewater from food processing plants (3, 4). The type strain, CBS5640/NRRL Y-1871, was originally cultured by Wickerham in 1942 and has since been isolated from the fermentation of alcoholic beverages (5, 6), sugarcane (7), and from clinical settings as a pathogen (8–10).

Interestingly, the isolate reported from a premature infant was resistant to amphotericin B and itraconazole (8). In another case, antifungal treatment of an adult with a *C. fabianii* infection led to variants immune to fluconazole and voriconazole. Biofilm formation possibly played a role in its increased resistance (11). Thus, understanding the genomic capacity of this species could help treat infections. While this was only the third case of *C. fabianii* infection found in a neonate, it indicates uncommon fungi are potential health hazards. In this project, isolate YJS4271 was cultured from olives in Castilla la Mancha, Spain (12).

Genome sequencing was performed using the Illumina HiSeq2000 platform. We produced 280-bp insert libraries and generated ~75-fold coverage with 100-bp paired-end reads. In total 9,010,526 reads were obtained and trimmed according to quality criteria with cutadapt (13). The reads were assembled using SOAPdenovo2, version r240 (14), with a k-mer size of 75. The final assembly consisted of 1,481 contigs with an *N*_50_ length of 23,883 bp and a maximum length of 98,416 bp. The contigs were assembled into 419 scaffolds, revealing a genome of 12,362,755 bp. We performed annotation on 50 scaffolds, which were larger than 5 kb (cumulative size of 12,277,034 bp, with 44.4% G+C content). Based on the reference genomes of two closely related species, *Debaryomyces hansenii* and *Wickerhamomyces ciferrii* (15, 16), a total of 5,713 putative protein coding genes were found using the Amadea Annotation transfer tool (Isoft, France). Interestingly, 767 genes (12.9% of the genes) are interrupted by spliceosomal introns. *C. fabianii* is thus one of the most intron-rich species of the *Saccharomycotina* yeasts sequenced so far. Functional annotation was attributed to the coding sequences on the basis of protein similarity with *S. cerevisiae*. Coding sequences with no similarity to those in *S. cerevisiae* were annotated using the refseq database. We identified 139 tRNA across the scaffolds using tRNAscan-SE v1.3.1 (17).

The *C. fabianii* genome is approximately 12.3 Mb and contains 5,944 putative protein coding genes. Based on whole-genome comparison, *C. fabianii* is closely related to *W. ciferrii*. Nevertheless, the genome content is very different from *W. ciferri*, which is 15.9 Mb with 6,702 protein-coding genes (16). Additionally, the average G+C content is 30.4%, much lower than 44.4% in *C. fabianii*. The genome of *Cyberlindnera fabianii* will provide a foundation for future assessment of this species as a pathogen and lend insight into its role in biotechnological applications including fermentation and wastewater processing.

### Nucleotide sequence accession numbers.

The sequence of the *C. fabianii* genome has been deposited at European Nucleotide Archive under the accession numbers LK052886–LK052935.
